# Endovascular Repair of Ascending Aortic Pseudoaneurysm (Zone 0) After Coronary Artery Bypass Grafting

**DOI:** 10.1016/j.jaccas.2025.103637

**Published:** 2025-06-04

**Authors:** Jonathan J. Szeto, Jason J. Han, Amit Iyengar, Rohan Shad, Chase R. Brown

**Affiliations:** a Perelman School of Medicine, University of Pennsylvania, Philadelphia, Pennsylvania, USA; b Division of Cardiovascular Surgery, University of Pennsylvania, Philadelphia, Pennsylvania, USA

**Keywords:** coronary artery bypass graft complication, pseudoaneurysm, thoracic endovascular aortic repair

## Abstract

Aortic pseudoaneurysms, rare but potentially life-threatening complications following coronary artery bypass grafting (CABG), are ideally addressed with re-exploration and open repair. We present a 67-year-old female who had recently undergone a CABG and developed a large mediastinal pseudoaneurysm from the site of the proximal saphenous vein graft anastomosis but was at prohibitive risk for redo open surgery due to significant comorbidities. An application of current endovascular devices was required to treat this post-CABG complication. Thoracic endovascular aortic repair of the ascending aorta was effective in treating the aortic pseudoaneurysm CABG complication in this patient who was at prohibitive risk of redo open surgery.

## Graphical Abstract

**Figure:**
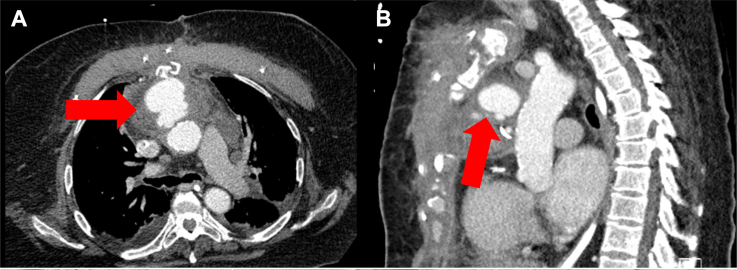

## Case summary

**Figure 1:**
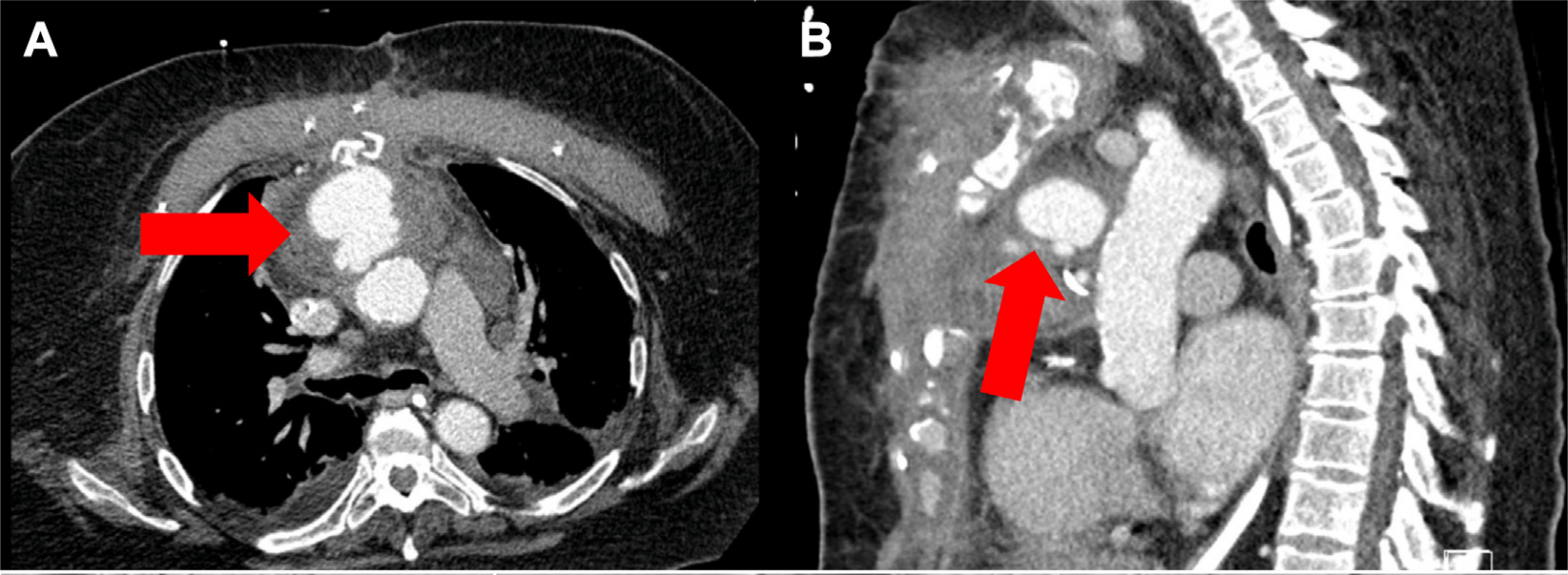
Preoperative Contrast Enhanced Computed Tomography Angiography Preoperative contrast-enhanced computed tomography angiography shows the ascending aorta zone 0 pseudoaneurysm (5 cm × 5 cm × 5 cm) arising in the saphenous vein graft anastomosis in both axial (A) and sagittal (B) views.

**Figure 2:**
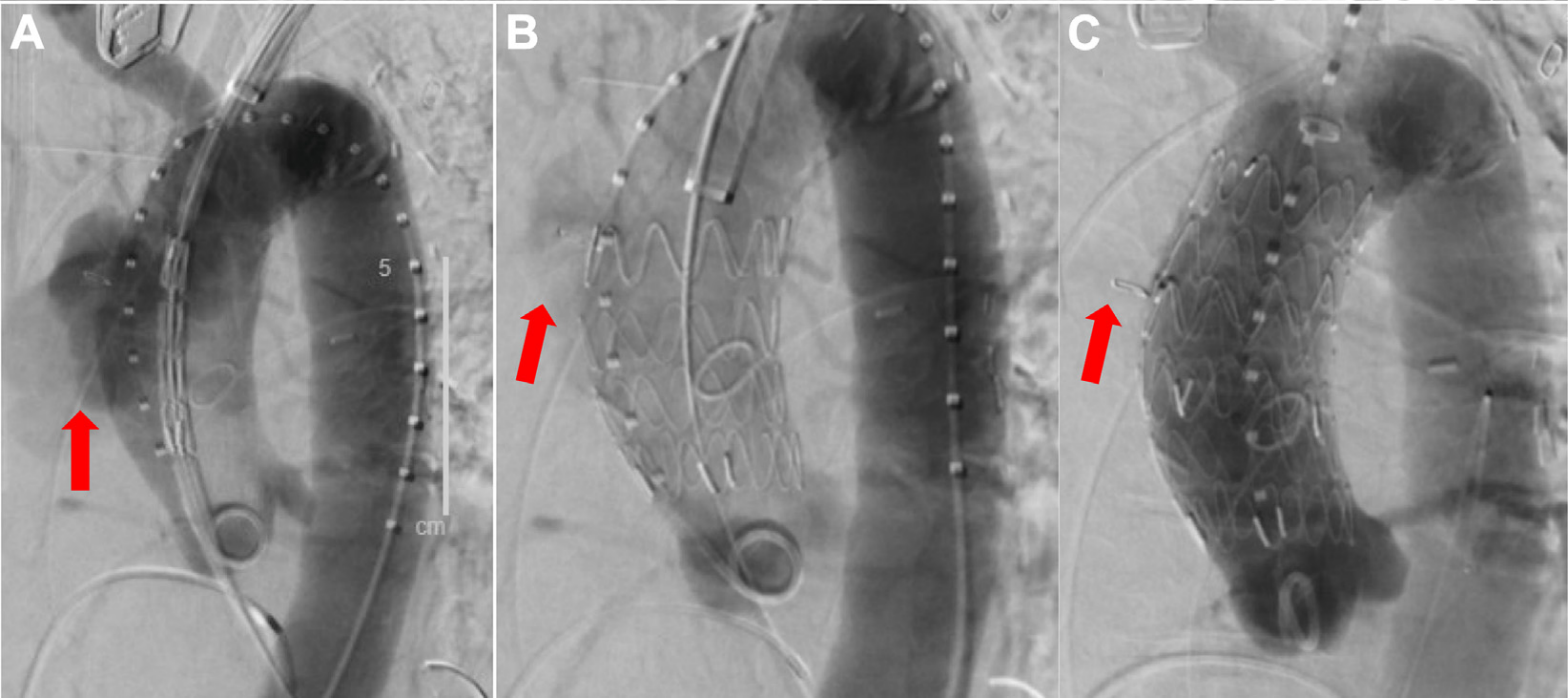
Intraoperative Fluoroscopic Angiography Pseudoaneurysm (red arrow) before stenting (A), after inserting first proximal endovascular aortic repair endograft (EVAR) stent (B), and after inserting the second distal EVAR endograft stent to ensure complete exclusion (C). Insertion of the second EVAR stent led to full exclusion of the pseudoaneurysm.

A 67-year-old female who recently underwent a coronary artery bypass graft involving bypassing 2 blocked coronary arteries (left internal mammary artery–left anterior descending artery and saphenous vein graft [SVG]–obtuse marginal) presented with worsening symptoms of weakness, dyspnea, peripheral edema, and chest pain during the same admission. A computed tomography angiogram was performed that showed a large mediastinal pseudoaneurysm arising from the site of the proximal SVG anastomosis on the ascending aorta (Figures 1 and 2). Given her severe chronic obstructive pulmonary disease, stage 3 chronic kidney disease, and prior sternal wound infection requiring pectoralis flap closure, the patient was deemed prohibitively high risk for redo open surgery and underwent an endovascular repair (Video 1).

**Take-Home Message**

• TEVAR of the ascending aorta can be a useful tool to address aortic complications arising from a coronary artery bypass graft procedure in patients who are at prohibitive risk of redo open surgery.

## Discussion

Aortic pseudoaneurysms are rare but potentially life-threatening complications following coronary artery bypass graft. Dehiscence at the proximal anastomosis, in part due to technical failure, can lead to rupturing of the aortic wall, causing a pseudoaneurysm. Although this clinical complication is ideally addressed with re-exploration and open repair, some patients are deemed prohibitively high risk for open surgery.^1^ Because of the lack of a U.S. Food and Drug Administration–approved endograft designed to treat ascending aortic pathology, creative application of current endovascular devices was required to prevent further growth or rupture of the pseudoaneurysm in this patient with suitable aortic anatomy. Medical or palliative care could be considered if the patient was not fit for an endovascular intervention; however, these approaches have poor prognoses.

Exclusion of the SVG–obtuse marginal anastomosis had the potential to cause acute myocardial ischemia. Thus, it was imperative to evaluate the patient’s coronary anatomy preoperatively via a left heart catheterization study (Video 2) and ensure bailout options that would protect against life-threatening myocardial infarction. In this case, we proceeded because the patient had adequate collateralization from her native left main coronary artery and the patent left internal mammary artery–left anterior descending artery graft. If this had not been the case, we considered stenting the lesion in her left main coronary artery before thoracic endovascular aortic repair (TEVAR) or simply tolerating a lateral wall myocardial infarction. Postoperatively, an electrocardiogram suggested no evidence of coronary ischemia.

Postoperative computed tomography imaging revealed a small type II endoleak into the pseudoaneurysm sac (Supplemental Figure 1). Before the operation, the team acknowledged the potential for type II endoleak due to retrograde flow from the open SVG. Preoperative embolism of the SVG graft was considered; however, the team did not proceed because there was no way to safely embolize the SVG. Half of type II endoleaks resolve spontaneously and persistent type II endoleaks are not associated with decreased survival, so the team decided that the risk of complications from an unsuccessful embolization outweighed the potential complications of a type II endoleak.^2^ Zone 0 TEVARs are especially challenging due to various anatomical factors, such as aortic arch angulation, supra-aortic head vessel branching, and high blood flow.^3^ The difficult anatomy of the aortic arch, combined with a lack of specific devices designed for zone 0 TEVAR, limit this procedure from becoming more standard fashion. Ongoing investigation of ascending TEVAR endograft technology must still be fully evaluated; however, this case shows that they can be a useful tool in managing postoperative ascending aorta complications.

## Funding Support and Author Disclosures

The authors have reported that they have no relationships relevant to the contents of this paper to disclose.

## Appendix

Supplemental Figure 1

Video 1 Intraoperative Fluoroscopy Intraoperative CT fluoroscopy illustrates deployment of EVAR devices into the ascending aorta and exclusion of the zone 0 pseudoaneurysm.

Video 2 Preoperative Left Heart Catheterization Left heart catheterization demonstrating patent grafts and pseudoaneurysm on the proximal segment.
