# Supplementary material for: Endovascular Repair of Ascending Aortic Pseudoaneurysm (Zone 0) After Coronary Artery Bypass Grafting
Source: JACC Case Rep. 2025 Jun 4;30(13):103637. doi: 10.1016/j.jaccas.2025.103637 (PMC12235162; doi:10.1016/j.jaccas.2025.103637)
Supplement: Supplemental Figure 1 [file mmc1.docx]

**Supplemental Figure 1:** **Postoperative Computed Tomography Demonstrating Zone 0 Aortic Pseudoaneurysm Repair**

Postoperative axial view of computed tomography angiography showing thrombosed pseudoaneurysm after EVAR procedure with minor type II endoleak into the pseudoaneurysm sac.
